# IFN-γ blockade after genetic inhibition of PD-1 aggravates skeletal muscle damage and impairs skeletal muscle regeneration

**DOI:** 10.1186/s11658-023-00439-8

**Published:** 2023-04-04

**Authors:** Shuzhao Zhuang, Aaron Russell, Yifan Guo, Yingying Xu, Weihua Xiao

**Affiliations:** 1 Shanghai Frontiers Science Research Base of Exercise and Metabolic Health, Shanghai University of Sport, Shanghai, China; 2 Key Laboratory of Exercise and Health Sciences, Shanghai University of Sport, Ministry of Education, Shanghai, China; 3 Institute for Physical Activity and Nutrition, School of Exercise and Nutrition Sciences, Deakin University, Geelong, Australia

**Keywords:** Skeletal muscle, Macrophage, Neutrophil, PD-1, IFN-γ

## Abstract

**Background:**

Innate immune responses play essential roles in skeletal muscle recovery after injury. Programmed cell death protein 1 (PD-1) contributes to skeletal muscle regeneration by promoting macrophage proinflammatory to anti-inflammatory phenotype transition. Interferon (IFN)-γ induces proinflammatory macrophages that appear to hinder myogenesis in vitro. Therefore, we tested the hypothesis that blocking IFN-γ in PD-1 knockout mice may dampen inflammation and promote skeletal muscle regeneration via regulating the macrophage phenotype and neutrophils.

**Methods:**

Anti-IFN-γ antibody was administered in PD-1 knockout mice, and cardiotoxin (CTX) injection was performed to induce acute skeletal muscle injury. Hematoxylin and eosin (HE) staining was used to view morphological changes of injured and regenerated skeletal muscle. Masson’s trichrome staining was used to assess the degree of fibrosis. Gene expressions of proinflammatory and anti-inflammatory factors, fibrosis-related factors, and myogenic regulator factors were determined by real-time polymerase chain reaction (PCR). Changes in macrophage phenotype were examined by western blot and real-time PCR. Immunofluorescence was used to detect the accumulation of proinflammatory macrophages, anti-inflammatory macrophages, and neutrophils.

**Results:**

IFN-γ blockade in PD-1 knockout mice did not alleviate skeletal muscle damage or improve regeneration following acute cardiotoxin-induced injury. Instead, it exacerbated skeletal muscle inflammation and fibrosis, and impaired regeneration via inhibiting macrophage accumulation, blocking macrophage proinflammatory to anti-inflammatory transition, and enhancing infiltration of neutrophils.

**Conclusion:**

IFN-γ is crucial for efficient skeletal muscle regeneration in the absence of PD-1.

**Supplementary Information:**

The online version contains supplementary material available at 10.1186/s11658-023-00439-8.

## Introduction

Acute skeletal muscle injury is a common form of various sports injuries [1]. Immediately following the injury, blood-borne neutrophils infiltrate the damaged muscle by rolling, adhesion, and transendothelial migration. This neutrophil invasion is detected within the first hour post injury, with numbers peaking 24–48 h post injury and concentrations remaining elevated for up to 5 days at the injury site [2]. Infiltrating neutrophils may have contrasting effects on postinjury skeletal muscle events. On the one hand, invading neutrophils are capable of removing cellular debris produced by damaged tissue through releasing proteases. On the other hand, they can amplify the inflammatory reaction via the secretion of various proinflammatory cytokines, such as interleukin-6 (IL-6) and tumor necrosis factor-alpha (TNF-α) [3]. Circulating neutrophils can be recruited to the injury site by chemotaxis to generate a positive feedback loop, thereby expanding the inflammatory response [4]. Neutrophil-secreted factors, such as macrophage inflammatory protein 1-alpha (MIP-1α) and matrix metalloproteinase-9 (MMP-9), promote the proliferation of myogenic precursor cells (MPCs), yet prolonged presence of these cytokines impedes MPC differentiation and maturation [5].

Initiation of neutrophil-driven inflammatory damage is hampered by tissue-resident macrophages through the inhibition of a feedforward chemoattractant signaling cascade [6]. Neutrophil-secreted chemotactic factors, such as IL-1 and IL-8, recruit monocyte-derived macrophages to the injured muscle [3]. Classically, macrophages are classified into two phenotypically and functionally distinct groups on the basis of acting stimuli: classically activated (proinflammatory) macrophage (M1) and alternatively activated (anti-inflammatory) macrophage (M2). M1 polarization is elicited by T helper type 1 (Th1) cytokines such as interferon-γ (IFN-γ) and TNF-α, or lipopolysaccharide (LPS) [7]. M1 macrophages exert a powerful cytotoxic response by producing IL-6, TNF-α, and nitric oxide; the latter regarded among the most dependable markers for M1 macrophage activation [8]. M2 phenotypical differentiation is activated by protolerogenic T helper type 2 (Th2)-derived cytokines, such as IL-4 and IL-13, during the recovery process after injury [9]. M2 macrophages directly contribute to inflammation resolution and tissue healing by production of cytokines, such as IL-10 and transforming growth factor-β (TGF-β) [10].

IFN-γ, as the sole member of type II interferon, is a pleiotropic regulatory protein in immunological cell signaling [11]. A wide range of immune cells, including activated T lymphocytes, group 1 innate lymphoid cells (ILC1s), and natural killer cells, produce IFN-γ [12]. IFN-γ is a potent activator of pro-inflammatory macrophages and neutrophils, which exacerbate muscle damage after acute injury [13]. IFN-γ drives the expression of M1 macrophage genes, while inhibiting genes involved in the expression of M2 macrophage [14]. IFN-γ also upregulates the responsiveness of neutrophils to chemokines, thereby improving their invasiveness [15]. IFN-γ promotes myoblast proliferation but inhibits myogenic differentiation in vitro, as indicated by reduced myosin heavy chain content [16]. Thus, dysregulated IFN-γ expression has the potential to expand inflammatory response and impair muscle regeneration.

Programmed cell death protein 1 (PD-1, also known as CD279) is a member of the CD28 superfamily. It is a 288 amino acid type-I transmembrane protein, widely induced on T cells, natural killer cells, B cells, and myeloid cells upon activation [17, 18]. When PD-1 binds to its ligands, programmed cell death ligand 1 (PD-L1, also known as CD274) or PD-L2 (also known as CD273), in the presence of T-cell receptor (TCR) complex, it elicits inhibitory signals modulating antigen receptor signaling, peripheral tolerance, and immune-mediated tissue damage [19]. In the tumor microenvironment, IFN-γ upregulates the expression of PD-L1 on tumor cells, protecting them from T-cell-mediated elimination, resulting in immune evasion [20]. The application of anti-PD-1 monoclonal antibodies is widely used in clinical practice to help patients prevent cancer cells. However, anti-PD-1 therapy has complications, such as myopathy, with frequent involvement of extraocular and bulbar muscles [21]. Other research also showed low skeletal muscle mass is related to reduced survival in cancer patients treated with PD-1/PDL-1 checkpoint inhibitors [22, 23]. We have already found that PD-1 promotes skeletal muscle regeneration through regulating macrophage polarization [24]. IFN-γ production is enhanced in skeletal muscle after hindlimb ischemia in PD-1 knockout mice, which contributes to increased tissue inflammation, abnormal angiogenesis and delayed muscle regeneration [25]. On the basis of these results, we further hypothesize that IFN-γ blockade after genetic deletion of PD-1 reduces inflammation and promotes muscle regeneration through the regulation of macrophage phenotypes and neutrophils. Hence, we tested the regulatory effects of PD-1 and IFN-γ on macrophages and neutrophils in a cardiotoxin (CTX)-induced skeletal muscle injury model. The data revealed that PD-1 and IFN-γ are essential for modulating muscle inflammation and promoting muscle regeneration.

## Materials and methods

### Animals

**Fig. 1:**
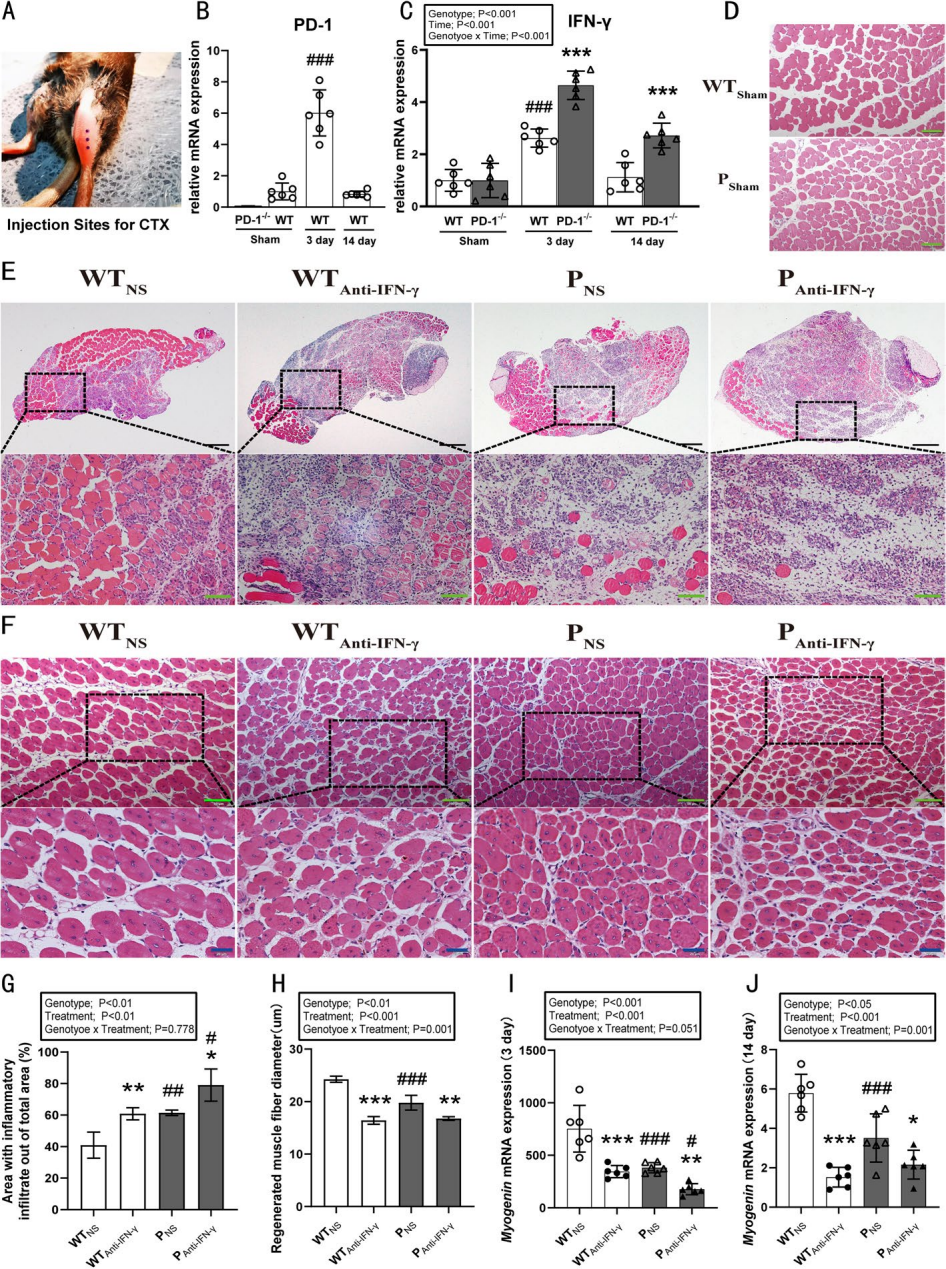
PD-1 knockout and IFN-γ blockade aggravate muscle damage and impair muscle regeneration. WT_Sham_: wild-type uninjured group, P_Sham_: PD-1^−/−^ uninjured group, WT_NS_: wild-type injured group with normal saline injection, WT_Anti-IFN-γ_: wild-type injured group with anti-IFN-γ antibody injection, P_NS_: PD-1^−/−^ injured group with normal saline injection, P_Anti-IFN-**γ**_: PD-1^−/−^ injured group with anti-IFN-γ antibody injection. **A** Injection sites for CTX. **B** PD-1 mRNA expression in wild-type mice after skeletal muscle injury. **C** IFN-γ mRNA expression in wild-type mice and PD-1^−/−^ mice after skeletal muscle injury. (***P < 0.001, different from WT mice at the same timepoint, different from each other and the PD-1^−/−^ sham mice. ^###^*P* < 0.001, different from WT sham and WT 14-day groups). **D** hematoxylin and eosin (HE) staining of uninjured tibialis anterior (TA) muscle of mice of both genotypes. **E** HE staining of injured TA muscle of mice of both genotypes injected with CTX along with either anti-IFN-γ antibody or normal saline at 3-days post injury. **F** HE staining of injured TA muscle of mice of both genotypes injected with CTX, along with either anti-IFN-γ antibody or normal saline at 14 days post injury. **G** Quantification of area with inflammatory infiltration as in **E**. **H** Quantification of regenerated muscle fiber diameter as in **F**, **I** myogenin mRNA expression at 3 days post injury, **J** myogenin mRNA expression at 14 days post injury. *P < 0.05, **P < 0.01, ***P < 0.001, different from NS treatment when comparing the same genotype. ^#^*P* < 0.05, ^##^*P* < 0.01, ^###^*P* < 0.001, different from WT mice when comparing the same treatment. Data are shown as mean ± standard deviation (SD), *n* = 6 (**B**, **C**, **I**, **J**), *n* = 3 (**G**, **H**). Black scale bar, 200 μm; green scale bar, 50 μm; blue scale bar, 20 μm

Thirty wild-type (WT) C57BL/6 mice were obtained from the Shanghai Model Organisms Center. Thirty PD-1^−/−^ mice on the C57BL/6 background (B6.Cg-Pdcd1tm1.1Shr/J) were provided by Jackson Laboratory. Female mice at 8 weeks of age were administered CTX (BOYAO, 9012–91–3) as illustrated in Fig. 1A. InVivoMAb anti-mouse IFN-γ (Bio X Cell, Clone: XMG1.2) was used to neutralize IFN-γ. Mice were maintained at room temperature (23 ± 2 °C) with a humidity of 55.6 ± 4%, a 12:12 h light–dark cycle, and free access to water and pellet food.

Thirty wild-type C57BL/6 mice were randomly divided into three groups: a wild-type uninjured group (WT_Sham_, *n* = 6), a wild-type injured group with normal saline injection (WT_NS_, *n* = 12), and a wild-type injured group with anti-IFN-γ antibody injection (WT_Anti-IFN-γ_, *n* = 12). Thirty PD-1^−/−^ mice were randomly divided into three groups: a PD-1^−/−^ uninjured group (P_Sham_, *n* = 6), a PD-1^−/−^ injured group with normal saline injection (P_NS_, *n* = 12), and a PD-1^−/−^ injured group with anti-IFN-γ antibody injection (P_Anti-IFN-γ_, *n* = 12). Mice in the injured groups were randomly selected for analyses at two timepoints: 3 days and 14 days post injury, with six mice per group per time. All experimental procedures were approved by the Animal Care and Use Committee at Shanghai University of Sport in China.

### Generation of muscle injury and neutralization of IFN-γ

Mice were anesthetized using 2–5% isoflurane and subsequently maintained under a light dose (1.5–2%) on a heating pad. Fur was removed from the skin surface of the tibialis anterior (TA) muscle by shaving, and subsequent application of a mild depilatory cream. To increase the precision of the operation and homogeneity of injury, three points with a distance of 0.5 cm were marked on the surface of the TA with a marking pen to indicate injection sites. After cleaning the injection sites with alcohol, 30 µl of 20 µM CTX was injected intramuscularly into the TA muscle by three separate injections (10 µl per injection), resulting in significant myofiber myolysis. Immediately after CTX administration, mice were either intraperitoneally injected with 200 µg InVivoMAb anti-mouse IFN-γ in 200 µl InVivoPure pH 8.0 Dilution Buffer (Bio X cell, IP0080) for in vivo blocking of IFN-γ, or with equivalent normal saline (NS) as a control. The dose of anti-IFN-γ antibody was based on previous publications in which the action of IFN-γ was successfully blocked in mice [26, 27]. Anti-IFN-γ antibody was administered once per day in the 3-day postinjury groups and every second day in the 14-day postinjury groups. TA muscles were harvested at 3 and 14 days after injury for further analyses.

### Histological staining

Paraffin specimens were prepared from the left TA muscle for hematoxylin and eosin (HE) staining and Masson’s trichrome staining, as previously described [28, 29]. Four micrometer cross-sections were cut from the mid-belly of each TA muscle and stained with HE to reveal the general muscle architecture. Muscle damage was evaluated by area containing inflammatory infiltrate. Regenerating muscle fibers were identified by the presence of centrally located nuclei. Masson’s trichrome staining was used to assess the degree of fibrosis. Percentage of fibrosis area/cross-sectional area was calculated to assess fibrosis formation. Morphological analysis was performed with ImageJ software.

### Immunofluorescence

Immunofluorescence methods were performed on paraffin-embedded sections of the TA muscles fixed with 4% paraformaldehyde as previously described [24]. After deparaffinization, heat-induced antigen retrieval in sodium citrate antigen-repair solution, and blocking with 10% donkey serum at room temperature for 2 h, sections were incubated overnight at 4 °C with primary antibodies against macrophage antigen-2 (Mac-2, Cedarlane, CL8942AP, dilution 1:1000), inducible nitric oxide synthase (iNOS, Abcam, ab15323, dilution 1:100), arginase1 (Arg1, Cell Signaling Technology, 93668S, dilution 1:100), and lymphocyte antigen 6 complex locus G6D (Ly-6G, AbD Serotec, MCA771GA, dilution 1:100). After washing, Alexa Fluor 555 (Abcam, ab150154, dilution 1:500) and Alexa Fluor 488 (Abcam, ab150073, dilution 1:500) secondary antibodies were applied for 1 h at room temperature in the dark. After washing, the sections were counterstained with DAPI, rinsed, sealed with a cover slip, followed by analysis using a confocal microscope (Zeiss LSM70). For each muscle sample, at least five random fields of view were analyzed. The number of positive cells was counted with ImageJ software.

### Western blotting

Protein was extracted from the TA muscle using RIPA lysis buffer containing PMSF (Beyotime Biotechnology, ST506) and a protease inhibitor (Beyotime Biotechnology, P1050) as previously described, followed by homogenization [30]. Protein concentration was determined using the bicinchoninic acid (BCA) protein assay kit (Beyotime Biotechnology, P0010). Protein samples containing equal amounts of protein were electrophoresed on a 10% SDS–polyacrylamide gel then transferred to polyvinylidene fluoride (PVDF) membranes. After blocking with 5% non-fat milk powder in Tris-buffered saline containing 0.1% Tween 20 (TBST), membranes were incubated with antibodies against Mac-2 (Cedarlane, CL8942AP, dilution 1:2000) and α-tubulin (Proteintec, 11224-1-AP, dilution 1:1000) at 4 °C overnight, followed by incubation with appropriate horseradish peroxidase (HRP)-conjugated secondary antibodies at room temperature for 1 h. Alpha tubulin is commonly used as a control protein for various tissues, as well as for highlighting the structure of cytoskeleton. It has previously been used in a large quantity of skeletal muscle research [31, 32]. Specific bands were visualized with an enhanced chemiluminescence (ECL) kit and quantified by densitometry.

### RNA extraction and real-time polymerase chain reaction (PCR)

Total RNA was extracted from the right TA muscle using TRIzol (Invitrogen), as previously described [33]. RNA samples were reverse transcribed into cDNA using the PrimeScript RT Reagent Kit (TaKaRa Bio, RR037A), according to the manufacturer’s instructions, followed by quantitative real-time PCR utilizing 10 µl 2× Maxima SYBR Green/ROX qPCR Master Mix (Vazyme Biotech), 1 µl cDNA, 8.2 µl nuclease-free water, and 300 nM of each primer on a StepOnePlus Real-Time PCR-Cycler (Life Technologies). Amplification was performed using the following parameters: activation at 95 °C for 10 min, followed by 40 cycles of denaturation at 95 °C for 15 s, and annealing/extension at 60 °C for 60 s. The primers used were designed and synthesized commercially (Sangon Biotech) (Table 1). The relative mRNA expression was calculated by the 2^−ΔΔCT^ method.

**Table 1:** Primer sequences for real-time PCR

Target gene	Forward primer sequences	Reverse primer sequences
PD-1	5′-GCCACCTTCACCTGCAGCTTGT-3′	5′-AAACCGGCCTTCTGGTTTGGGC-3′
IFN-γ	5′-GCTTTGCAGCTCTTCCTCAT-3′	5′-GTCACCATCCTTTTGCCAGT-3′
IL-1β	5′-TGACGTTCCCATTAGACAACTG-3′	5′-CCGTCTTTCATTACACAGGACA-3′
TNF-α	5′-CTTCTGTCTACTGAACTTCGGG-3′	5′-CACTTGGTGGTTTGCTACGAC-3'
IL-6	5′-GAACAACGATGATGCACTTGC-3′	5′-CTTCATGTACTCCAGGTAGCTATGGT-3′
IL-10	5′-GCTCTTACTGACTGGCATGAG-3′	5′-CGCAGCTCTAGGAGCATGTG-3′
Myogenin	5′-CCAGTACATTGAGCGCCTAC-3′	5′-ACCGAACTCCAGTGCATTGC-3′
TGF-β	5′-TGCGCTTGCAGAGATTAAAA-3'	5′- CGTCAAAAGACAGCCACTCA-3′
Col3a1	5′-GTCCACGAGGTGACAAAGGT-3′	5′-GATGCCCACTTGTTCCATCT-3′
Mac-2	5′-CAGGACAGGCTCCTCCTAGTGC-3′	5′-CCAGCAGCAGGATAGCCTCCAG-3′
F4/80	5′-AACATGCAACCTGCCACAAC-3′	5′-TTCACAGGATTCGTCCAGGC-3′
CD68	5′-CAAAGCTTCTGCTGTGGAAAT-3′	5′-GACTGGTCACGGTTGCAAG-3′
iNOS	5′-CTGCAGCACTTGGATCAG-3′	5′-CGTACCAGGCCCAATGAG-3′
CD86	5′-AGTGATCGCCAACTTCAGTGAACC-3′	5′-GGTGACCTTGCTTAGACGTGCAG-3′
Arg1	5′-GAACACGGCAGTGGCTTTAAC-3′	5′-TGCTTAGCTCTGTCTGCTTTGC-3′
CD206	5′-GGATTGTGGAGCAGATGGAAG -3′	5′-CTTGAATGGAAATGCACAGAC-3′
GAPDH	5′-ACTCCACTCACGGCAAATTC-3′	5′-TCTCCATGGTGGTGAAGACA-3′

### Statistical analysis

Data were analyzed using SPSS 26.0 and presented as mean ± standard deviation. One-way or two-way ANOVA was used to analyze statistical data. Bonferroni correction method was used as a post hoc test. The significant levels were set at *(#)*P* < 0.05, **(##)*P* < 0.01, ***(###)*P* < 0.001.

## Results

### PD-1 knockout and IFN-γ blockade aggravate muscle damage and impair muscle regeneration

PD-1 expression was not detected in PD-1^−/−^ mice (Fig. 1B). One-way ANOVA indicated a significant difference in PD-1 mRNA levels between the sham injury group and the 3 day and 14 day injured groups (*P* < 0.001). Post hoc analysis revealed that PD-1 mRNA expression increased approximately sixfold 3 days post injury, but had returned to the basal levels observed in the sham injury group 14 days post injury (Fig. 1B). When measuring changes in IFN-γ mRNA expression, two-way ANOVA indicated a genotype × time interaction (*P* < 0.001), as well as main effects for genotype (*P* < 0.001) and time (*P* < 0.001) (Fig. 1C). Post hoc analyses indicated that IFN-γ mRNA expression in WT mice and PD-1^−/−^ injured mice were increased 3 days post injury (*P* < 0.001), with the increase greater in the PD-1^−/−^ mice (*P* < 0.001). At day 14 post injury, IFN-γ mRNA expression levels in the WT mice had returned to baseline; however, they remained elevated above baseline in the PD-1^−/−^ injured mice (*P* < 0.001).

HE staining indicated inflammatory cell accumulation, muscle fiber swelling, and necrosis in all injured groups, 3 days after muscle injury (Fig. 1E). No obvious differences were found in muscle morphology between uninjured muscles from the WT_Sham_ group and the P_Sham_ group (Fig. 1D). There were main effects for genotype (*P* < 0.01) and treatment (*P* < 0.01) on the areas of inflammatory infiltrate after injury (Fig. 1G). The area with inflammatory cell infiltration in the WT_Anti-IFN-γ_ (*P* < 0.01) and P_NS_ group (*P* < 0.01) were significantly larger than in the WT_NS_ group (control). Anti-IFN-γ treatment in the PD-1^−/−^ mice increased areas of necrosis and inflammatory infiltrate (*P* < 0.05). However, there was no significant interaction between IFN-γ blockade and PD-1 knockout. These results indicate that both IFN-γ blockade and PD-1 knockout aggravate skeletal muscle injury, while the combined effects lead to further aggravation of muscle injury.

HE staining revealed robust regenerating muscle fibers with centrally located nuclei in all injured groups by day 14 post injury (Fig. 1F). A genotype × time interaction (*P* = 0.001), together with main effects for genotype (*P* < 0.01) and treatment (*P* < 0.001), were found for regenerated muscle fiber diameter (Fig. 1H). Post hoc analyses revealed that regenerated muscle fiber diameter in the WT_Anti-IFN-γ_ and P_NS_ groups were significantly smaller than in the WT_NS_ group (*P* < 0.001). Anti-IFN-γ treatment in the PD-1^−/−^ mice decreased regenerated muscle fiber diameter when compared with P_NS_ group (*P* < 0.01). When measuring myogenin mRNA expression, two-way ANOVA indicated a genotype × time interaction (14 day: *P* = 0.001), as well as main effects for genotype (3 days: *P* < 0.001, 14 days: *P* < 0.05) and treatment (both *P* < 0.001) (Fig. 1I, J). Post hoc analysis revealed that myogenin mRNA levels in the WT_Anti-IFN-γ_ and P_NS_ group were significantly lower than in the WT_NS_ group (*P* < 0.001) at both 3 and 14 days post injury. Anti-IFN-γ treatment in the PD-1^−/−^ mice decreased myogenin mRNA expression compared with P_NS_ (3 days: *P* < 0.01, 14 days: *P* < 0.05) and WT_Anti-IFN-γ_ groups (3 days: *P* < 0.05). These results revealed that blocking IFN-γ after PD-1 knockout further delays the regenerative process.

### PD-1 knockout and IFN-γ blockade exacerbate inflammation and fibrosis of injured skeletal muscle

**Fig. 2:**
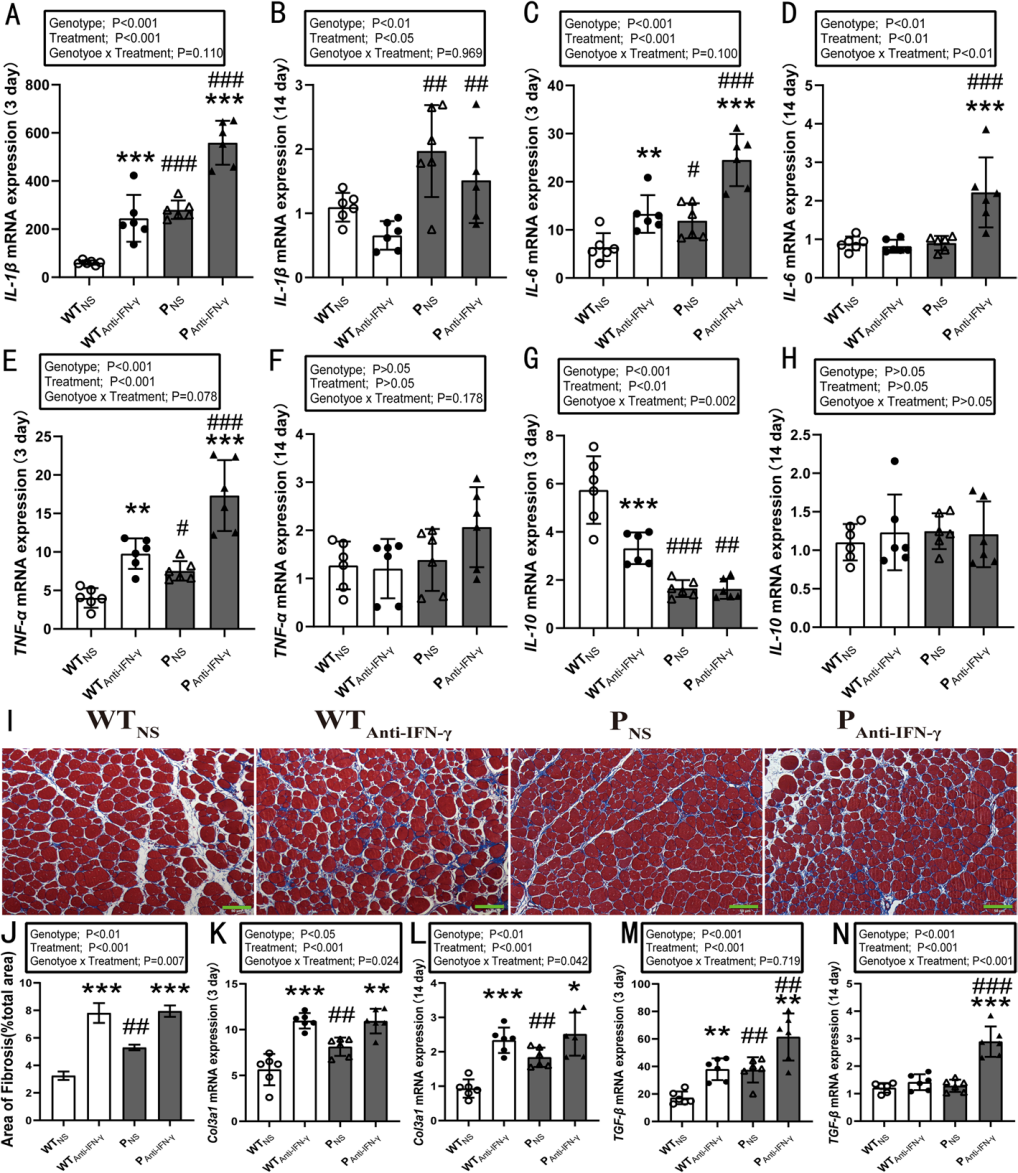
PD-1 knockout and IFN-γ blockade exacerbate inflammation and fibrosis of injured skeletal muscle. WT_NS_: wild-type injured group with normal saline injection, WT_Anti-IFN-γ_: wild-type injured group with anti-IFN-γ antibody injection, P_NS_: PD-1^−/−^ injured group with normal saline injection, P_Anti-IFN-γ_: PD-1^−/−^ injured group with Anti-IFN-γ antibody injection. **A** IL-1β mRNA expression at 3 days post injury, **B** IL-1β mRNA expression at 14 days post injury, **C** IL-6 mRNA expression at 3 days post injury, **D** IL-6 mRNA expression at 14 days post injury, **E** TNF-α mRNA expression at 3 days post injury, **F** TNF-α mRNA expression at 14 days post injury, **G** IL-10 mRNA expression at 3 days post injury; **H** IL-10 mRNA expression at 14 days post injury, **I** Masson’s trichrome staining of injured TA muscle of mice of both genotypes injected with CTX, along with either anti-IFN-γ antibody or normal saline at 14 days post injury, **J** Quantification of area of fibrosis as in **I**, **K** Col3a1 mRNA expression at 3 days post injury, **L** Col3a1 mRNA expression at 14 days post injury, **M** TGF-β mRNA expression at 3 days post injury, **N** TGF-β mRNA expression at 14 days post injury. *P < 0.05, **P < 0.01, ***P < 0.001, different from NS treatment when comparing the same genotype. ^#^*P* < 0.05, ^##^*P* < 0.01, ^###^*P* < 0.001, different from WT mice when comparing the same treatment. Data are shown as mean ± standard deviation (SD), *n* = 6 (**A**–**H**, **K**–**N**), *n* = 3 (**J**). Green scale bar, 50 μm

The mRNA expression levels of proinflammatory cytokines IL-1β, IL-6 and TNF-α increased substantially at 3 days post injury (Fig. 2A, C, E). Two-way ANOVA indicated main effects for genotype (*P* < 0.001) and treatment (*P* < 0.001). Post hoc analyses showed IL-1β, IL-6, and TNF-α mRNA expression levels in the WT_Anti-IFN-γ_ (IL-1β: *P* < 0.001, IL-6: *P* < 0.01, TNF-α: *P* < 0.01) and P_NS_ groups (IL-1β: *P* < 0.001, IL-6: *P* < 0.05, TNF-α: *P* < 0.05) were significantly higher than in the WT_NS_ group. Anti-IFN-γ treatment in the PD-1^−/−^ mice increased IL-1β, IL-6, and TNF-α mRNA expression levels (all *P* < 0.001). At 14 days post injury, two-way ANOVA revealed main effects for genotype (IL-1β: *P* < 0.01, IL-6: *P* < 0.001) and treatment (IL-1β: *P* < 0.05, IL-6: *P* < 0.001) (Fig. 2B, D). Post hoc analyses showed that IL-1β and IL-6 levels in the P_Anti-IFN-γ_ group were significantly higher than in the WT_Anti-IFN-γ_ group (IL-1β: *P* < 0.01, IL-6: *P* < 0.001). Moreover, anti-IFN-γ treatment in the PD-1^−/−^ mice increased IL-6 mRNA expression compared with P_NS_ group (*P* < 0.001) (Fig. 2D). At 3 days post injury, an interaction between genotype and treatment (*P* = 0.002), together with main effects for genotype (*P* < 0.001) and treatment (*P* < 0.01) on anti-inflammatory cytokine IL-10 mRNA expression (Fig. 2G) was observed. IL-10 mRNA expression in the WT_Anti-IFN-γ_ (*P* < 0.001) and P_NS_ groups (*P* < 0.001) were significantly lower than in the WT_NS_ group. IFN-γ blockade in the PD-1^−/−^ mice decreased IL-10 mRNA expression compared with WT_Anti-IFN-γ_ group (*P* < 0.01). However, no significant difference in IL-10 mRNA expression was observed 14 days post injury in any of the groups (*P* > 0.05) (Fig. 2H). These results suggest that IFN-γ blockade or PD-1 knockout exacerbates injured muscle inflammation, while the combined effects further induce a stronger pro-inflammatory reaction contributing to the muscle injury.

Following Masson staining, analyses of the amount of fibrosis showed a genotype × time interaction (*P* = 0.007), together with main effects for genotype (*P* < 0.01) and treatment (*P* < 0.001) (Fig. 2I, J). The fibrotic area in the WT_Anti-IFN-γ_ (*P* < 0.001) and P_NS_ groups (*P* < 0.01) were significantly larger than in the WT_NS_ group. Blocking the IFN-γ protein in the PD-1^−/−^ mice increased the area of fibrosis when compared with knocking out PD-1 alone (*P* < 0.001).

As for the mRNA expression level of Col3a1, a significant interaction between genotype and treatment was observed (3 days: *P* = 0.024, 14 days: *P* = 0.042), as well as a main effect for genotype (3 days: *P* < 0.05, 14 days: *P* < 0.01) and treatment (both *P* < 0.001) (Fig. 2K, L). The Col3a1 mRNA levels in the WT_Anti-IFN-γ_ (*P* < 0.001) and P_NS_ groups (*P* < 0.01) were significantly larger than in the WT_NS_ group at both 3 and 14 days post injury. Anti-IFN-γ treatment in the PD-1^−/−^ mice increased Col3a1 mRNA expression compared with P_NS_ group (3 days: *P* < 0.01, 14 days: *P* < 0.05).

With respect to TGF-β mRNA expression, a main effect for genotype (*P* < 0.001) and treatment (*P* < 0.001) at both 3 and 14 days post injury (Fig. 2M, N) was observed. TGF-β mRNA levels in the WT_Anti-IFN-γ_ (*P* < 0.01) and P_NS_ groups (*P* < 0.01) were significantly higher than in the WT_NS_ group at 3 days post injury. A further significant increase in TGF-β mRNA levels in the P_Anti-IFN-γ_ was observed when compared with the WT_Anti-IFN-γ_ (3 days: *P* < 0.01, 14 days: *P* < 0.001) and P_NS_ groups (3 days: *P* < 0.01, 14 days: *P* < 0.001). These results demonstrate that IFN-γ blockade or PD-1 knockout aggravates muscle fibrosis, while the combined effects are more significant than PD-1 knockout alone.

### Effects of PD-1 knockout and IFN-γ blockade on macrophage and neutrophil infiltration following muscle injury

**Fig. 3:**
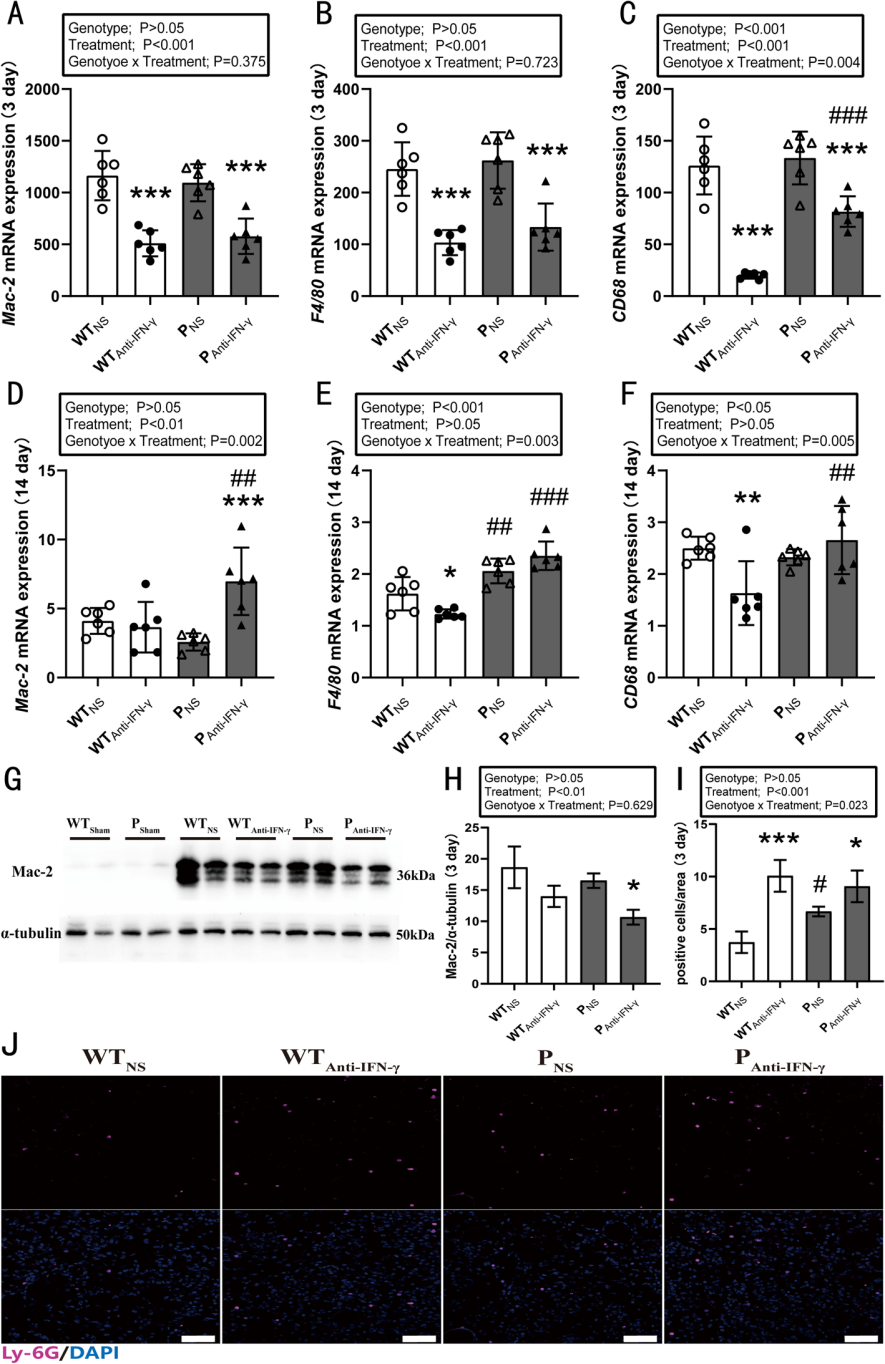
Effects of PD-1 knockout and IFN-γ blockade on macrophage and neutrophil infiltration following injury. WT_Sham_: wild-type uninjured group, P_Sham_: PD-1^−/−^ uninjured group, WT_NS_: wild-type injured group with normal saline injection, WT_Anti-IFN-γ_: wild-type injured group with anti-IFN-γ antibody injection, P_NS_: PD-1^−/−^ injured group with normal saline injection, P_Anti-IFN-γ_: PD-1^−/−^ injured group with anti-IFN-γ antibody injection. **A** Mac-2 mRNA expression at 3 days post injury, **B** F4/80 mRNA expression at 3 days post injury, **C** CD68 mRNA expression at 3 days post injury, **D** Mac-2 mRNA expression at 14 days post injury, **E** F4/80 mRNA expression at 14 days post injury, **F** CD68 mRNA expression at 14 days post injury, **G** protein expression levels of Mac-2 and internal control α-tubulin at 3 days post injury, **H** quantification of Mac-2 expression as in **G**, **I** quantification of neutrophils per field as in **J**, **J** representative immunofluorescence staining of Ly-6G at 3 days post injury. *P < 0.05, **P < 0.01, ***P < 0.001, different from NS treatment when comparing the same genotype. ^#^*P* < 0.05, ^##^*P* < 0.01, ^###^*P* < 0.001, different from WT mice when comparing the same treatment. Data are shown as mean ± standard deviation (SD), *n* = 6 (**A**–**F**), *n* = 4 (**H**), *n* = 3 (**I**). White scale bar, 50 μm

At 3 days post injury, a main effect of anti-IFN-γ treatment on Mac-2, F4/80, and CD68 mRNA expression levels was observed (*P* < 0.001) (Fig. 3A–C). A main effect for genotype (*P* < 0.001) and an interaction between genotype and treatment (*P* = 0.004) were observed for CD68 mRNA expression (Fig. 3C). Post hoc analyses indicated blocking IFN-γ protein decreased Mac-2, F4/80, and CD68 mRNA expression in both WT and PD-1^−/−^ injured mice (*P* < 0.001). At 14 days post injury, an interaction between genotype and treatment on Mac-2 (*P* = 0.002), F4/80 (*P* = 0.003), and CD68 (*P* = 0.005) mRNA expression was observed (Fig. 3D–F). Anti-IFN-γ treatment decreased F4/80 (*P* < 0.05) and CD68 mRNA expression (*P* < 0.01) in the WT mice. Anti-IFN-γ treatment in the PD-1^−/−^ mice increased Mac-2 (*P* < 0.01), F4/80 (*P* < 0.001), and CD68 mRNA expression (*P* < 0.01) when compared with the WT_Anti-IFN-γ_ group.

Mac-2 protein levels increased significantly in all injured groups at 3 days post injury (Fig. 3G). There was a main effect of anti-IFN-γ treatment (*P* < 0.01) (Fig. 3H) with Mac-2 protein levels decreasing in all treated groups (*P* < 0.05). These results suggest that IFN-γ blockade inhibits macrophage infiltration following injury, but this effect was abolished at late stage (14 days) of muscle injury in the PD-1^−/−^ mice.

At 3 days post injury, a significant infiltration of neutrophils in injured muscle was observed (Fig. 3I, J). When comparing the number of neutrophils, a main effect for treatment (*P* < 0.001) and genotype × treatment interaction (*P* = 0.023) were observed. Post hoc analyses showed that the number of neutrophils in the WT_Anti-IFN-γ_ (*P* < 0.001) and P_NS_ groups (*P* < 0.05) was greater than in the WT_NS_ group. Anti-IFN-γ treatment increased the number of neutrophils in the PD-1^−/−^ mice when compared with P_NS_ group (*P* < 0.05). The results showed that PD-1 knockout and IFN-γ blockade promote neutrophil infiltration, but the combined effect is not greater than blocking IFN-γ alone.

### Effects of PD-1 knockout and IFN-γ blockade on proinflammatory macrophages

**Fig. 4:**
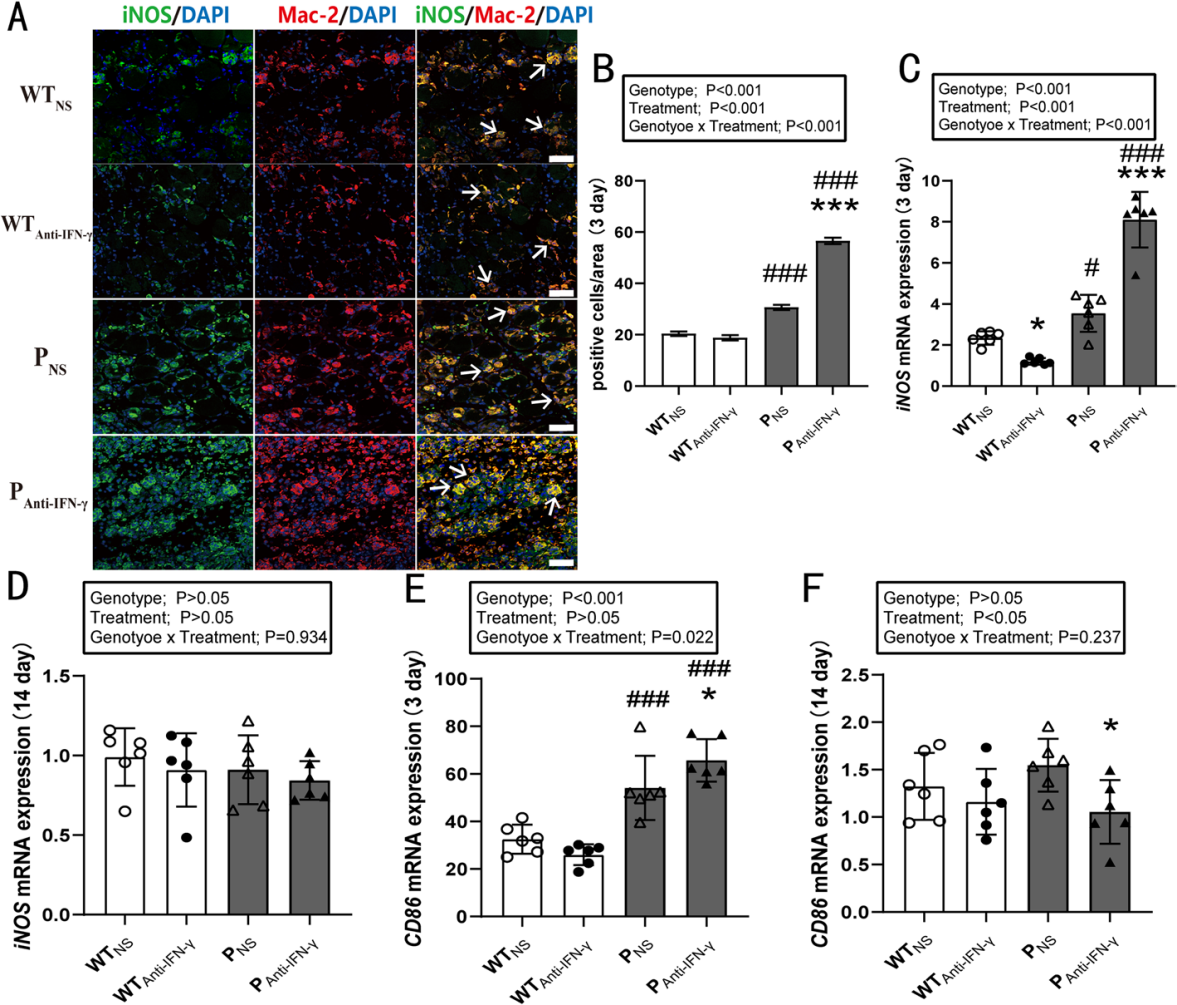
IFN-γ blockade after PD-1 knockout increases proinflammatory macrophage activation in injured skeletal muscle. WT_NS_: wild-type injured group with normal saline injection, WT_Anti-IFN-γ_: wild-type injured group with Anti-IFN-γ antibody injection, P_NS_: PD-1^−/−^ injured group with normal saline injection, P_Anti-IFN-γ_: PD-1^−/−^ injured group with Anti-IFN-γ antibody injection. **A** Representative double immunofluorescence staining of iNOS and Mac-2 at 3 days post injury, **B** quantification of pro-inflammatory macrophages per field as in **A**, **C** iNOS mRNA expression at 3 days post injury, **D** iNOS mRNA expression at 14 days post injury, **E** CD86 mRNA expression at 3 days post injury, **F** CD86 mRNA expression at 14 days post injury. *P < 0.05, ***P < 0.001, different from NS treatment when comparing the same genotype. ^#^*P* < 0.05, ^###^*P* < 0.001, different from WT mice when comparing the same treatment. Data are shown as mean ± standard deviation (SD), *n* = 3 (**B**), *n* = 6 (**C**–**F**). White scale bar, 50 μm. → Proinflammatory macrophage

At 3 days post injury, two-way ANOVA indicated a genotype × time interaction (*P* < 0.001), as well as main effects for genotype (*P* < 0.001) and treatment (*P* < 0.001) on the number of proinflammatory macrophages (Fig. 4A, B). Post hoc analysis revealed that the number of proinflammatory macrophages in the P_Anti-IFN-γ_ group was significantly higher than in the WT_Anti-IFN-γ_ (*P* < 0.001) and P_NS_ groups (*P* < 0.001). The number of proinflammatory macrophages in the P_NS_ group was larger than in the WT_NS_ group (*P* < 0.001). At 14 days post injury, there was no proinflammatory macrophage infiltration in any group (data not shown).

For iNOS mRNA expression, a genotype × time interaction (*P* < 0.001) and main effect for genotype (*P* < 0.001) and treatment (*P* < 0.001) at 3 days post injury was observed. (Fig. 4C). Anti-IFN-γ treatment in the WT mice decreased iNOS mRNA expression (*P* < 0.05), while PD-1 knockout increased iNOS mRNA expression (*P* < 0.05). The genotype × time interaction highlighted a further significant increase in iNOS mRNA expression in the P_Anti-IFN-γ_ group when compared with the WT_Anti-IFN-γ_ (*P* < 0.001) and P_NS_ groups (*P* < 0.001). For CD86 mRNA expression, a genotype × time interaction (*P* = 0.022) and main effect for genotype (*P* < 0.001) at 3 days post injury was observed (Fig. 4E). PD-1 knockout increased CD86 mRNA levels (*P* < 0.001). Furthermore, the genotype × time interaction indicated a significant increase in CD86 mRNA expression in the P_Anti-IFN-γ_ group compared with the WT_Anti-IFN-γ_ (*P* < 0.001) and P_NS_ groups (*P* < 0.05). Blocking PD-1 decreased CD86 mRNA levels in PD-1^−/−^ mice 14 days post injury (main effect of treatment, *P* < 0.05) (Fig. 4F). These results suggest that PD-1 knockout increases M1-type macrophage activation, while blocking IFN-γ further promotes this response.

### Effects of PD-1 knockout and IFN-γ blockade on anti-inflammatory macrophages

**Fig. 5:**
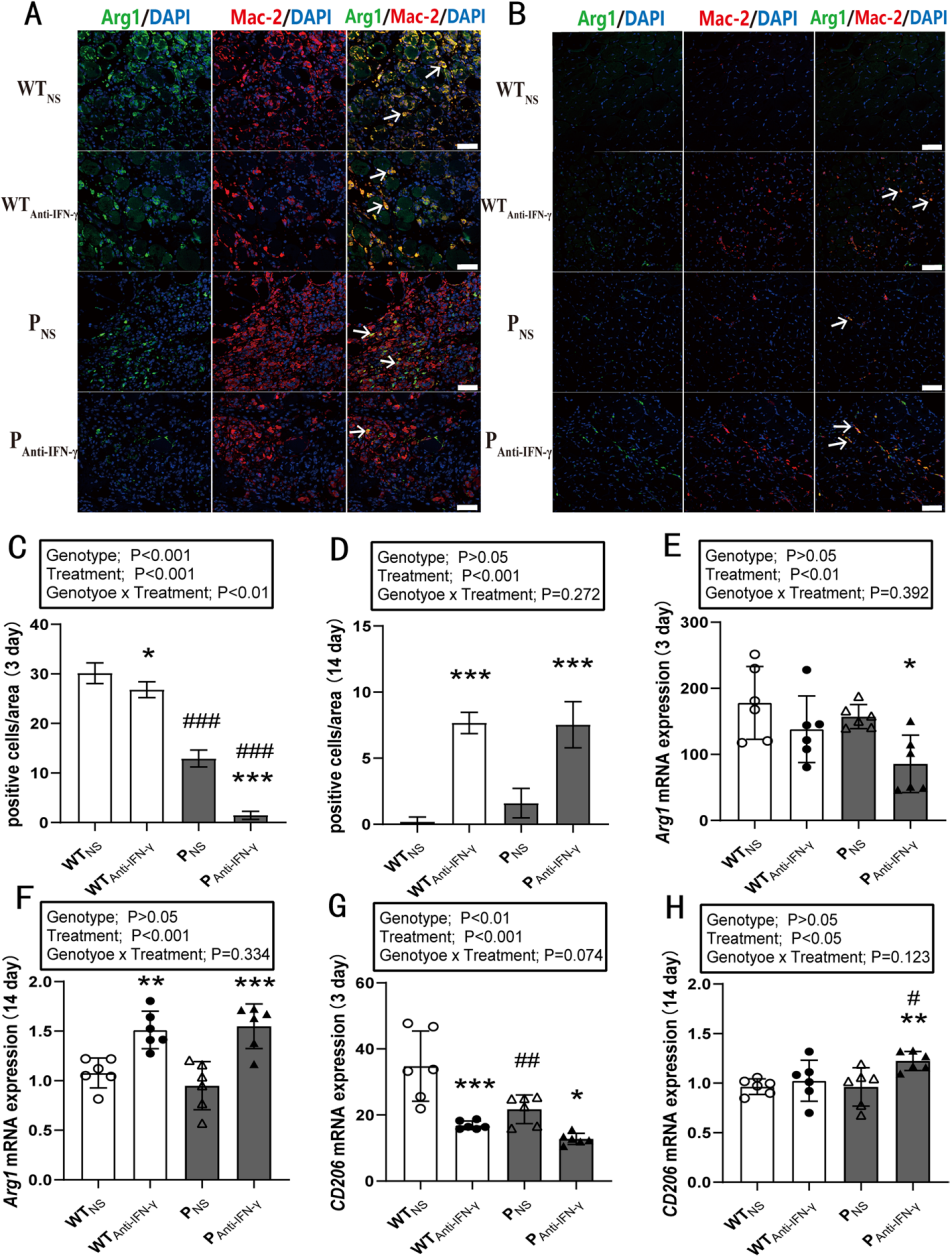
IFN-γ blockade after PD-1 knockout reduces anti-inflammatory macrophage activation in injured skeletal muscle. WT_NS_: wild-type injured group with normal saline injection, WT_Anti-IFN-γ_: wild-type injured group with Anti-IFN-γ antibody injection, P_NS_: PD-1^−/−^ injured group with normal saline injection, P_Anti-IFN-γ_: PD-1^−/−^ injured group with Anti-IFN-γ antibody injection. **A** Representative double immunofluorescence staining of Arg1 and Mac-2 at 3 days post injury, **B** representative double immunofluorescence staining of Arg1 and Mac-2 at 14 days post injury, **C** quantification of anti-inflammatory macrophages per field as in **A**, **D** quantification of anti-inflammatory macrophages per field as in **B**, **E** Arg1 mRNA expression at 3 days post injury, **F** Arg1 mRNA expression at 14 days post injury, **G** CD206 mRNA expression at 3 days post injury, **H** CD206 mRNA expression at 14 days post injury. *P < 0.05, **P < 0.01, ***P < 0.001, different from NS treatment when comparing the same genotype. ^#^*P* < 0.05, ^##^*P* < 0.01, ^###^*P* < 0.001, different from WT mice when comparing the same treatment. Data are shown as mean ± standard deviation (SD), *n* = 3 (**C**, **D**), *n* = 6 (**E**–**H**). White scale bar, 50 μm. → Anti-inflammatory macrophage

Two-way ANOVA indicated a genotype × time interaction (*P* < 0.01), as well as main effects for genotype (*P* < 0.001) and treatment (*P* < 0.001) on the number of anti-inflammatory macrophages when measured 3 days post injury (Fig. 5A, C). Post hoc analysis indicated that the number of anti-inflammatory macrophages in the WT_Anti-IFN-γ_ (*P* < 0.05) and P_NS_ group (*P* < 0.001) was significantly lower than in the WT_NS_ group. Anti-IFN-γ treatment in the PD-1^−/−^ mice further decreased the number of anti-inflammatory macrophages (*P* < 0.001), as well as Arg1 (*P* < 0.05) and CD206 mRNA expression (*P* < 0.05) when compared with the P_NS_ group (Fig. 5C, E, G). CD206 mRNA expression in the WT_Anti-IFN-γ_ (*P* < 0.001) and P_NS_ group (*P* < 0.01) was significantly lower than in the WT_NS_ group (Fig. 5G).

Immunofluorescence analysis showed that anti-IFN-γ treatment increased the number of anti-inflammatory macrophages in both WT (*P* < 0.001) and PD-1^−/−^ mice (*P* < 0.001) when measured 14 days post injury (Fig. 5B, D). Two-way ANOVA indicated main effects for treatment on Arg1 (*P* < 0.001) and CD206 (*P* < 0.05) mRNA expression (Fig. 5F, H). Blocking IFN-γ protein increased Arg1 mRNA expression in WT (*P* < 0.01) and PD-1^−/−^ mice (*P* < 0.001) (Fig. 5F). Blocking IFN-γ protein in PD-1^−/−^ mice increased CD206 mRNA expression when compared with the WT_Anti-IFN-γ_ (*P* < 0.05) and P_NS_ groups (*P* < 0.01) (Fig. 5H). These results suggest that PD-1 knockout inhibits M2-type macrophage activation, while blocking IFN-γ delays M2-type macrophage activation.

## Discussion

In our present research, blocking the IFN-γ protein did not relieve muscle damage in PD-1 knockout or wild-type mice. On the contrary, blocking IFN-γ impaired muscle regeneration and exacerbated muscle fibrosis. Interestingly, blocking IFN-γ protein after genetic knockout of PD-1 dramatically increased inflammatory infiltration in injured skeletal muscle, when compared with blocking IFN-γ or knocking out PD-1 alone. Furthermore, IFN-γ blockade in PD-1 knockout mice further delayed muscle regeneration, when compared with knockout of PD-1 alone, following injury. Myogenin is a myogenic regulatory factor and controls the later stages of myogenic differentiation [34]. Our findings suggest that IFN-γ blockade in PD-1 knockout mice decreases *myogenin* expression, supporting observations that IFN-γ reduces myogenin protein level in *mdx* mice [35]. IFN-γ appears to attenuate myogenesis through direct inhibition of myogenin, and this IFN-γ-induced restriction is mediated by the class II major histocompatibility complex transactivator (CIITA) [36]. IFN-γ silences muscle-specific genes through the recruitment of polycomb repressive complex 2 (PRC2) and Jumonji family protein JARID2 to their promoters [37]. However, endogenous IFN-γ contributes to skeletal muscle regeneration [38]. IFN-γ upregulates the expression of major histocompatibility complex class II (MHC-II), intracellular adhesion molecule-1 (ICAM-1), and monocyte chemoattractant protein-1 (MCP-1) in muscle cells [39–41]. Due to this controversial phenomenon, deeper investigation into the mechanisms by which this occurs is required.

We confirmed that both IFN-γ blockade or PD-1 knockout enhanced muscle fibrosis, post injury. However, our previous work indicated that PD-1 knockout did not increase collagen fiber disposition in mechanical strike-induced injury of skeletal muscle. This difference may be a consequence of the more severe muscle damage caused by CTX than mechanical strike, which amplified the influence of PD-1 gene knockout. Our results also show that both IFN-γ blockade and PD-1 knockout significantly increased the levels of type III collagen alpha l (Col3a1) and TGF-β, while the combined effects further upregulated the gene expression of profibrotic cytokine, TGF-β, at both 3 and 14 days post injury. TGF-β signaling pathway acts as an important mediator for formation of collagen fibers [42]. Blocking cyclooxygenase-2 (COX-2) with drugs or siRNA suppresses 3-phosphoinositide-dependent protein kinase 1 (PDK1) expression and protein kinase B (AKT) phosphorylation induced by TGF-β stimulation, thereby reducing muscle fibrosis [43]. As a TGF-β signaling pathway inhibitor, IFN-γ reduces fibrosis formation in a laceration model [44]. IFN-γ null mice also exhibit enhanced tissue fibrosis when compared with wild-type mice. This indicates that IFN-γ exerts potent antifibrotic properties [38]. Col3a1 is an important component of the extracellular matrix (ECM) and is also a fibrillar forming collagen. miR-29, which is under negative regulation by TGF-β signaling, prohibits fibrogenic differentiation by inhibiting the expression of Col3a1 [45].

Inflammatory cells and cytokines play integral roles in the context of muscle damage. They help clear lytic and necrotic myofibers in the early period of muscle injury. However, their constant presence impairs regenerative processes. IL-1β, IL-6, TNF-α, and IL-10 all appear to regulate myogenesis. IL-1β stimulates myotube catabolism via activation of nuclear factor-κB (NF-κB) signaling, and elevation of atrogin-1/muscle atrophy F-box (atrogin-1/MAFbx) and muscle-specific RING-finger protein 1 (MuRF1) [46]. TNF-α at low concentrations promotes myogenesis, while high TNF-α concentrations inhibit myogenesis [9, 47, 48]. Both IL-1β and TNF-α appear to trigger the production of IL-6 by cultured myoblasts and myotubes [49–51]. IL-6 induces the proliferation of both C2C12 mouse myoblasts and primary human myoblasts at low and medium levels. However, high levels of this cytokine promote myogenic differentiation [52]. Also, chronically elevated production of IL-6 is closely associated with skeletal muscle wasting [53]. As an essential anti-inflammatory cytokine, IL-10 exerts its immunosuppressive effects by inhibiting proinflammatory factors, such as IL-1β and TNF-α [54]. We find here that either IFN-γ blockade or PD-1 knockout upregulates the expression of proinflammatory cytokines (IL-1β, IL-6, and TNF-α) and downregulates the expression of the anti-inflammatory cytokine IL-10 in the early phase of muscle injury. The results found in PD-1 knockout mice are similar to our previous findings in a muscle contusion injury model [24]. Further examination suggests that IFN-γ blockade after genetic knockout of PD-1 increases the expression of IL-1β, IL-6, and TNF-α at both early (3 days) and late (14 days) phases of muscle damage. This elevated proinflammatory status, combined with low anti-inflammatory action, may be the main culprit for poor muscle regeneration.

Macrophages play increasingly defined roles in the inflammatory response after skeletal muscle injury. Our observation that PD-1 knockout hinders the transition of the macrophage phenotype from proinflammatory to anti-inflammatory has been observed previously [55–58]. IFN-γ induces the M1 macrophage phenotype. However, blocking its expression exacerbates the inflammatory response and muscle damage in PD-1 knockout mice. Reasons for this unexpected phenomenon are largely unknown. To examine this further, we semiquantified the accumulation and subtypes of macrophages. We found that blocking IFN-γ decreases the gene expression of total macrophage markers Mac-2, F4/80, and CD68, which is consistent with the observation of reduced F4/80-positive macrophage accumulation in IFN-γ null mice [38]. However, PD-1 knockout did not significantly change the expression levels of Mac-2, F4/80, and CD68. This indicates that PD-1 may not influence total macrophage infiltration in injured muscle. Furthermore, IFN-γ blockade after PD-1 knockout significantly downregulated the gene expression of Mac-2, F4/80, and CD68, and also protein levels of Mac-2. This suggests that IFN-γ blockade also contributes to suppressing macrophage accumulation in PD-1 knockout mice.

To decipher the effects of PD-1 knockout and IFN-γ blockade on macrophage subtypes, we examined the accumulation of M1 and M2 macrophages in injured muscle. Blocking IFN-γ did not affect the number of M1 macrophages nor change the gene expression of CD86. However, it did reduce the number of M2 macrophages and gene expression of CD206. This suggests that IFN-γ blockade mainly inhibits the formation of M2 phenotype macrophages. In contrast, PD-1 knockout increased the number of M1 macrophages, as well as iNOS and CD86 gene expression, and decreased the number of M2 macrophages and CD206 gene expression. This suggests that PD-1 knockout promotes the M1 proinflammatory phenotype and also inhibits the M2 anti-inflammatory phenotype. This is consistent with our previous findings [24]. Moreover, IFN-γ blockade after PD-1 knockout further increased the number of M1 macrophages and iNOS and CD86 gene expression, and reduced the number of M2 macrophages and Arg1 and CD206 gene expression. Therefore, blocking IFN-γ after PD-1 knockout further promotes M1 macrophage activation and lowers M2 macrophage activation. Furthermore, blocking IFN-γ leads to significant M2 macrophage infiltration in both wild-type mice and PD-1 knockout mice at late stage (14 days) of muscle injury. Consistently blocking IFN-γ upregulates Arg1 and CD206 gene expression at 14 days post injury, indicating that IFN-γ blockade delays the infiltration of M2 macrophages.

Neutrophil recruitment and infiltration into the injured skeletal muscle plays a critical role in the mediation of local tissue damage. IFN-γ and TNF-α released by neutrophils delay myogenesis [59]. Neutrophils generate reactive oxygen species (ROS), which breaks down damaged muscle tissue and also regulates humoral autoimmunity via inhibition of IFN-γ [60, 61]. TNF-α and IL-1β also enhance IFN-γ receptor expression and augment neutrophil recruitment [62]. Furthermore, the PD-L1/PD-1 pathway participates in modulating direct neutrophil and tumor cell cross talk. Blockade of the interaction between PD-L1 and PD-1 increased tumor cell susceptibility to neutrophil cytotoxicity [63]. Our data indicate that both IFN-γ blockade and PD-1 knockout enhance neutrophil infiltration, which elicits a strong proinflammatory response and consequently exacerbates muscle injury. We also observed that the increased neutrophil infiltration caused by PD-1 knockout plus IFN-γ blockade is higher than that caused solely by knockout of PD-1, but is not different from that caused by IFN-γ blockade alone. It is hard to conclude if PD-1 knockout exerts effects in the absence of IFN-γ protein. Further examination is required to investigate neutrophil infiltration, and involved molecular pathways altered by PD-1 and IFN-γ, in the skeletal muscle in response to acute injury.

## Conclusion

In summary, blocking IFN-γ signaling after PD-1 knockout does not alleviate skeletal muscle damage or promote its regeneration. Instead, it exacerbates injured muscle inflammation, impairs muscle regeneration, and aggravates muscle fibrosis by inhibition of macrophage infiltration, blockade of macrophage proinflammatory to anti-inflammatory switching, and increased infiltration of neutrophils.

## Supplementary Information

**Additional file 1.** Original images of western blotting.

## Data Availability

The data used and/or analyzed during the current study are available from the corresponding author on reasonable request.

## Abbreviations

AKT: Protein kinase B
Arg1: Arginase1
CIITA: Class II major histocompatibility complex transactivator
Col3a1: Collagen type III alpha 1 chain
COX-2: Cyclooxygenase-2
CTX: Cardiotoxin
ECM: Extracellular matrix
ICAM-1: Intracellular adhesion molecule-1
IFN-γ: Interferon-γ
IL-6: Interleukin-6
iNOS: Inducible nitric oxide synthase
LPS: Lipopolysaccharide
Ly-6G: Lymphocyte antigen 6 complex locus G6D
M1: Proinflammatory macrophage
M2: Anti-inflammatory macrophage
Mac-2: Macrophage antigen-2
MAFbx: Muscle atrophy F-box
MCP-1: Monocyte chemoattractant protein-1
MHC-II: Major histocompatibility complex class II
MIP-1α: Macrophage inflammatory protein 1-alpha
MMP-9: Matrix metalloproteinase-9
MuRF1: Muscle-specific RING-finger protein 1
NF-κB: Nuclear factor-κB
NS: Normal saline
PD-1: Programmed cell death protein 1
PDK1: 3-Phosphoinositide-dependent protein kinase 1
PD-L1: Programmed cell death ligand 1
PRC2: Polycomb repressive complex 2
ROS: Reactive oxygen species
TCR: T-cell receptor
TGF-β: Transforming growth factor-β
Th1: T-helper type 1
TNF-α: Tumor necrosis factor-alpha

## References

[1] Hamilton B, Alonso JM, Best TM (2017). Time for a paradigm shift in the classification of muscle injuries. J Sport Health Sci.

[2] Tidball JG (2005). Inflammatory processes in muscle injury and repair. Am J Physiol Regul Integr Comp Physiol.

[3] Smith C, Kruger MJ, Smith RM, Myburgh KH (2008). The inflammatory response to skeletal muscle injury: illuminating complexities. Sports Med.

[4] Su WH, Wang CJ, Fu HC, Sheng CM, Tsai CC, Cheng JH (2019). Human Umbilical cord mesenchymal stem cells extricate bupivacaine-impaired skeletal muscle function via mitigating neutrophil-mediated acute inflammation and protecting against fibrosis. Int J Mol Sci.

[5] Seo BR, Payne CJ, McNamara SL, Freedman BR, Kwee BJ, Nam S (2021). Skeletal muscle regeneration with robotic actuation-mediated clearance of neutrophils. Sci Transl Med.

[6] Uderhardt S, Martins AJ, Tsang JS, Lämmermann T, Germain RN (2019). Resident macrophages cloak tissue microlesions to prevent neutrophil-driven inflammatory damage. Cell.

[7] Kloc M, Ghobrial RM, Wosik J, Lewicka A, Lewicki S, Kubiak JZ (2019). Macrophage functions in wound healing. J Tissue Eng Regen Med.

[8] De Santa F, Vitiello L, Torcinaro A, Ferraro E (2019). The role of metabolic remodeling in macrophage polarization and its effect on skeletal muscle regeneration. Antioxid Redox Signal.

[9] Scala P, Rehak L, Giudice V, Ciaglia E, Puca AA, Selleri C (2021). Stem cell and macrophage roles in skeletal muscle regenerative medicine. Int J Mol Sci.

[10] Funes SC, Rios M, Escobar-Vera J, Kalergis AM (2018). Implications of macrophage polarization in autoimmunity. Immunology.

[11] Fenimore J, Young HA (2016). Regulation of IFN-γ expression. Adv Exp Med Biol.

[12] Mock JR, Tune MK, Dial CF, Torres-Castillo J, Hagan RS, Doerschuk CM (2020). Effects of IFN-γ on immune cell kinetics during the resolution of acute lung injury. Physiol Rep.

[13] Tidball JG (2011). Mechanisms of muscle injury, repair, and regeneration. Compr Physiol.

[14] Novakovic B, Wang C, Logie C (2017). We can still be friends: IFN-γ breaks up macrophage enhancers. Immunity.

[15] Bonecchi R, Polentarutti N, Luini W, Borsatti A, Bernasconi S, Locati M (1999). Up-regulation of CCR1 and CCR3 and induction of chemotaxis to CC chemokines by IFN-gamma in human neutrophils. J Immunol.

[16] Grzelkowska-Kowalczyk K, Wicik Z, Majewska A, Tokarska J, Grabiec K, Kozłowski M (2015). Transcriptional regulation of important cellular processes in skeletal myogenesis through interferon-γ. J Interferon Cytokine Res.

[17] Nie M, Liu Y, Li XX, Min YN, Yang DD, Li Q (2019). PD-1/PD-L pathway potentially involved in ITP immunopathogenesis. Thromb Haemost.

[18] Yao A, Liu F, Chen K, Tang L, Liu L, Zhang K (2014). Programmed death 1 deficiency induces the polarization of macrophages/microglia to the M1 phenotype after spinal cord injury in mice. Neurotherapeutics.

[19] Wang Q, Xie B, Liu S, Shi Y, Tao Y, Xiao D (2021). What happens to the immune microenvironment after PD-1 inhibitor therapy?. Front Immunol.

[20] Chen X, Pan X, Zhang W, Guo H, Cheng S, He Q (2020). Epigenetic strategies synergize with PD-L1/PD-1 targeted cancer immunotherapies to enhance antitumor responses. Acta Pharm Sin B.

[21] Liewluck T, Kao JC, Mauermann ML (2018). PD-1 inhibitor-associated myopathies: emerging immune-mediated myopathies. J Immunother.

[22] Cortellini A, Bozzetti F, Palumbo P, Brocco D, Di Marino P, Tinari N (2020). Weighing the role of skeletal muscle mass and muscle density in cancer patients receiving PD-1/PD-L1 checkpoint inhibitors: a multicenter real-life study. Sci Rep.

[23] Takada K, Yoneshima Y, Tanaka K, Okamoto I, Shimokawa M, Wakasu S (2020). Clinical impact of skeletal muscle area in patients with non-small cell lung cancer treated with anti-PD-1 inhibitors. J Cancer Res Clin Oncol.

[24] Shou J, Shi X, Liu X, Chen Y, Chen P, Xiao W (2021). Programmed death-1 promotes contused skeletal muscle regeneration by regulating Treg cells and macrophages. Lab Invest.

[25] Liu X, Weng X, Xiao W, Xu X, Chen Y, Chen P (2021). Pharmacological and genetic inhibition of PD-1 demonstrate an important role of PD-1 in ischemia-induced skeletal muscle inflammation, oxidative stress, and angiogenesis. Front Immunol.

[26] Glasner A, Levi A, Enk J, Isaacson B, Viukov S, Orlanski S (2018). NKp46 receptor-mediated interferon-γ production by natural killer cells increases fibronectin 1 to alter tumor architecture and control metastasis. Immunity.

[27] Clever D, Roychoudhuri R, Constantinides MG, Askenase MH, Sukumar M, Klebanoff CA (2016). Oxygen sensing by T cells establishes an immunologically tolerant metastatic niche. Cell.

[28] Liu X, Zeng Z, Zhao L, Chen P, Xiao W (2019). Impaired skeletal muscle regeneration induced by macrophage depletion could be partly ameliorated by MGF injection. Front Physiol.

[29] Liu X, Zheng L, Zhou Y, Chen Y, Chen P, Xiao W (2019). BMSC Transplantation aggravates inflammation, oxidative stress, and fibrosis and impairs skeletal muscle regeneration. Front Physiol.

[30] Zheng L, Rao Z, Guo Y, Chen P, Xiao W (2020). High-intensity interval training restores glycolipid metabolism and mitochondrial function in skeletal muscle of mice with type 2 diabetes. Front Endocrinol.

[31] Li L, Liu H, Tao W, Wen S, Fu X, Yu S (2021). Pharmacological inhibition of HMGB1 prevents muscle wasting. Front Pharmacol.

[32] Liu Y, Liu P, Hu Y, Cao Y, Lu J, Yang Y (2021). Cold-induced RNA-binding protein promotes glucose metabolism and reduces apoptosis by increasing AKT phosphorylation in mouse skeletal muscle under acute cold exposure. Front Mol Biosci.

[33] Xiao W, Liu Y, Luo B, Zhao L, Liu X, Zeng Z (2016). Time-dependent gene expression analysis after mouse skeletal muscle contusion. J Sport Health Sci.

[34] Hernández-Hernández JM, García-González EG, Brun CE, Rudnicki MA (2017). The myogenic regulatory factors, determinants of muscle development, cell identity and regeneration. Semin Cell Dev Biol.

[35] Villalta SA, Deng B, Rinaldi C, Wehling-Henricks M, Tidball JG (2011). IFN-γ promotes muscle damage in the mdx mouse model of Duchenne muscular dystrophy by suppressing M2 macrophage activation and inhibiting muscle cell proliferation. J Immunol.

[36] Londhe P, Davie JK (2011). Gamma interferon modulates myogenesis through the major histocompatibility complex class II transactivator. CIITA Mol Cell Biol.

[37] Londhe P, Davie JK (2013). Interferon-γ resets muscle cell fate by stimulating the sequential recruitment of JARID2 and PRC2 to promoters to repress myogenesis. Sci Signal.

[38] Cheng M, Nguyen MH, Fantuzzi G, Koh TJ (2008). Endogenous interferon-gamma is required for efficient skeletal muscle regeneration. Am J Physiol Cell Physiol.

[39] Reyes-Reyna SM, Krolick KA (2000). Chemokine production by rat myocytes exposed to interferon-gamma. Clin Immunol.

[40] Stegall T, Krolick KA (2000). A monoclonal lewis rat myocyte line that responds to interferon-gamma: responsiveness with the potential to influence subsequent interactions with the immune system. Clin Immunol.

[41] Mantegazza R, Hughes SM, Mitchell D, Travis M, Blau HM, Steinman L (1991). Modulation of MHC class II antigen expression in human myoblasts after treatment with IFN-gamma. Neurology.

[42] Li K, Wang W, Xiao W (2023). Astaxanthin: a promising therapeutic agent for organ fibrosis. Pharmacol Res.

[43] Chen H, Qian Z, Zhang S, Tang J, Fang L, Jiang F (2021). Silencing COX-2 blocks PDK1/TRAF4-induced AKT activation to inhibit fibrogenesis during skeletal muscle atrophy. Redox Biol.

[44] Foster W, Li Y, Usas A, Somogyi G, Huard J (2003). Gamma interferon as an antifibrosis agent in skeletal muscle. J Orthop Res.

[45] Zhou L, Wang L, Lu L, Jiang P, Sun H, Wang H (2012). Inhibition of miR-29 by TGF-beta-Smad3 signaling through dual mechanisms promotes transdifferentiation of mouse myoblasts into myofibroblasts. PLoS ONE.

[46] Li W, Moylan JS, Chambers MA, Smith J, Reid MB (2009). Interleukin-1 stimulates catabolism in C2C12 myotubes. Am J Physiol Cell Physiol.

[47] O'Brien ME, Londino J, McGinnis M, Weathington N, Adair J, Suber T (2020). Tumor necrosis factor alpha regulates skeletal myogenesis by inhibiting SP1 interaction with cis-acting regulatory elements within the Fbxl2 gene promoter. Mol Cell Biol.

[48] Alvarez AM, DeOcesano-Pereira C, Teixeira C, Moreira V (2020). IL-1β and TNF-α modulation of proliferated and committed myoblasts: IL-6 and COX-2-derived prostaglandins as key actors in the mechanisms involved. Cells.

[49] Luo G, Hershko DD, Robb BW, Wray CJ, Hasselgren PO (2003). IL-1beta stimulates IL-6 production in cultured skeletal muscle cells through activation of MAP kinase signaling pathway and NF-kappa B. Am J Physiol Regul Integr Comp Physiol.

[50] Podbregar M, Lainscak M, Prelovsek O, Mars T (2013). Cytokine response of cultured skeletal muscle cells stimulated with proinflammatory factors depends on differentiation stage. ScientificWorldJournal.

[51] Otis JS, Niccoli S, Hawdon N, Sarvas JL, Frye MA, Chicco AJ (2014). Pro-inflammatory mediation of myoblast proliferation. PLoS ONE.

[52] Steyn PJ, Dzobo K, Smith RI, Myburgh KH (2019). Interleukin-6 induces myogenic differentiation via JAK2-STAT3 signaling in mouse C2C12 myoblast cell line and primary human myoblasts. Int J Mol Sci.

[53] Belizário JE, Fontes-Oliveira CC, Borges JP, Kashiabara JA, Vannier E (2016). Skeletal muscle wasting and renewal: a pivotal role of myokine IL-6. Springerplus.

[54] Lima AA, Spínola LG, Baccan G, Correia K, Oliva M, Vasconcelos JF (2014). Evaluation of corticosterone and IL-1β, IL-6, IL-10 and TNF-α expression after 670-nm laser photobiomodulation in rats. Lasers Med Sci.

[55] Dhupkar P, Gordon N, Stewart J, Kleinerman ES (2018). Anti-PD-1 therapy redirects macrophages from an M2 to an M1 phenotype inducing regression of OS lung metastases. Cancer Med.

[56] Rao G, Latha K, Ott M, Sabbagh A, Marisetty A, Ling X (2020). Anti-PD-1 induces M1 polarization in the glioma microenvironment and exerts therapeutic efficacy in the absence of CD8 cytotoxic T cells. Clin Cancer Res.

[57] Xiong H, Mittman S, Rodriguez R, Moskalenko M, Pacheco-Sanchez P, Yang Y (2019). Anti-PD-L1 treatment results in functional remodeling of the macrophage compartment. Cancer Res.

[58] Cai H, Zhang Y, Wang J, Gu J (2021). Defects in macrophage reprogramming in cancer therapy: the negative impact of PD-L1/PD-1. Front Immunol.

[59] Pizza FX, Peterson JM, Baas JH, Koh TJ (2005). Neutrophils contribute to muscle injury and impair its resolution after lengthening contractions in mice. J Physiol.

[60] Peake JM, Neubauer O, Della Gatta PA, Nosaka K (1985). Muscle damage and inflammation during recovery from exercise. J Appl Physiol.

[61] Huang X, Li J, Dorta-Estremera S, Di Domizio J, Anthony SM, Watowich SS (2015). Neutrophils regulate humoral autoimmunity by restricting interferon-γ production via the generation of reactive oxygen species. Cell Rep.

[62] Hackel A, Aksamit A, Bruderek K, Lang S, Brandau S (2021). TNF-α and IL-1β sensitize human MSC for IFN-γ signaling and enhance neutrophil recruitment. Eur J Immunol.

[63] Yajuk O, Baron M, Toker S, Zelter T, Fainsod-Levi T, Granot Z (2021). The PD-L1/PD-1 axis blocks neutrophil cytotoxicity in cancer. Cells.

