# Supplementary figures and images for: IFN-γ blockade after genetic inhibition of PD-1 aggravates skeletal muscle damage and impairs skeletal muscle regeneration
Source: Cell Mol Biol Lett. 2023 Apr 4;28:27. doi: 10.1186/s11658-023-00439-8 (PMC10071770; doi:10.1186/s11658-023-00439-8)

western blots of Mac-2:

1.


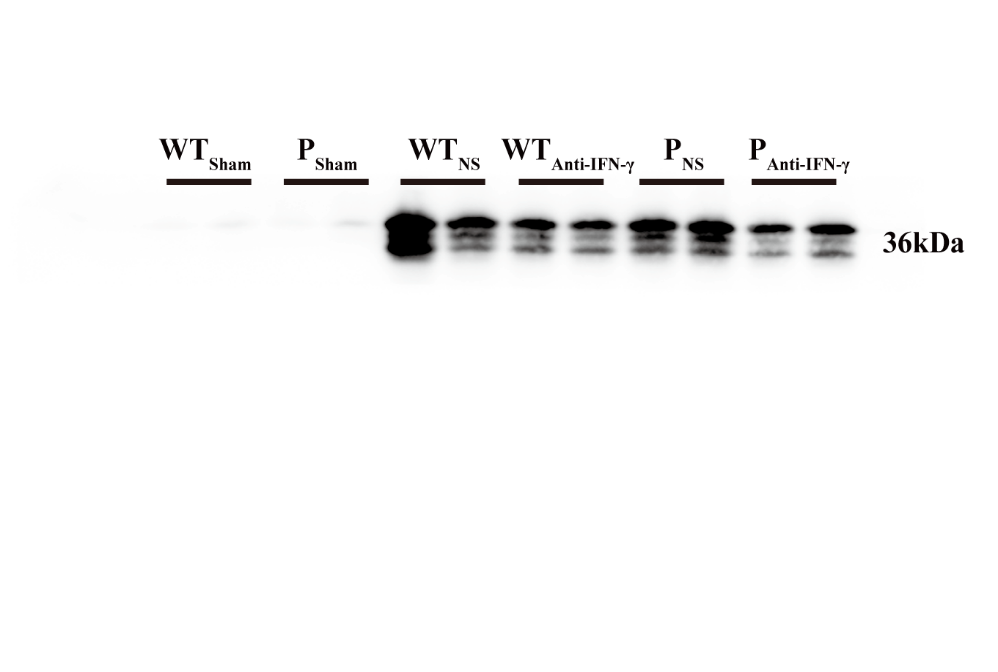


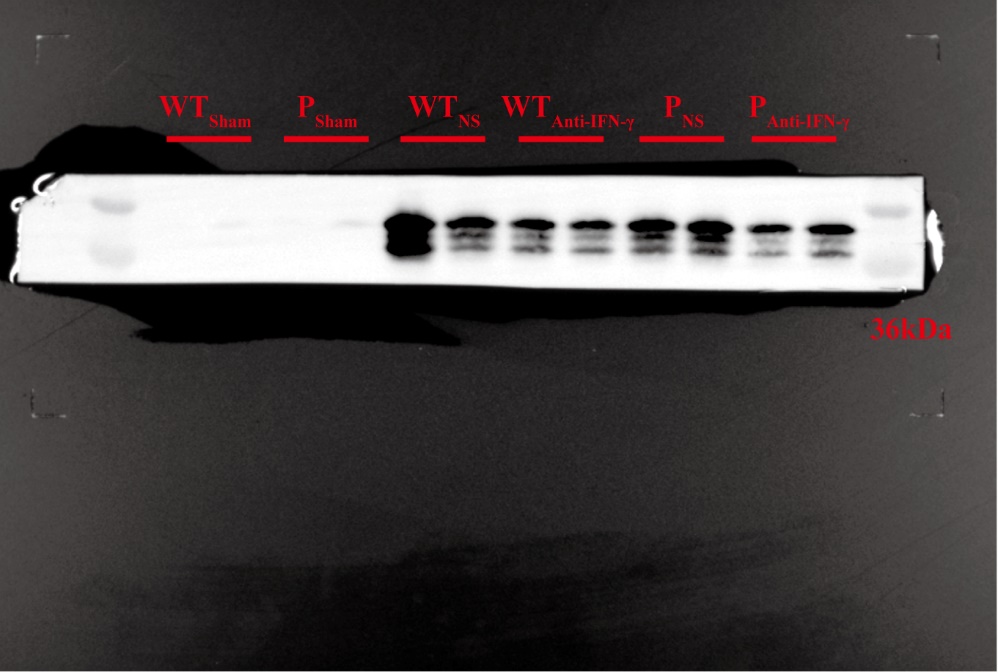


2.


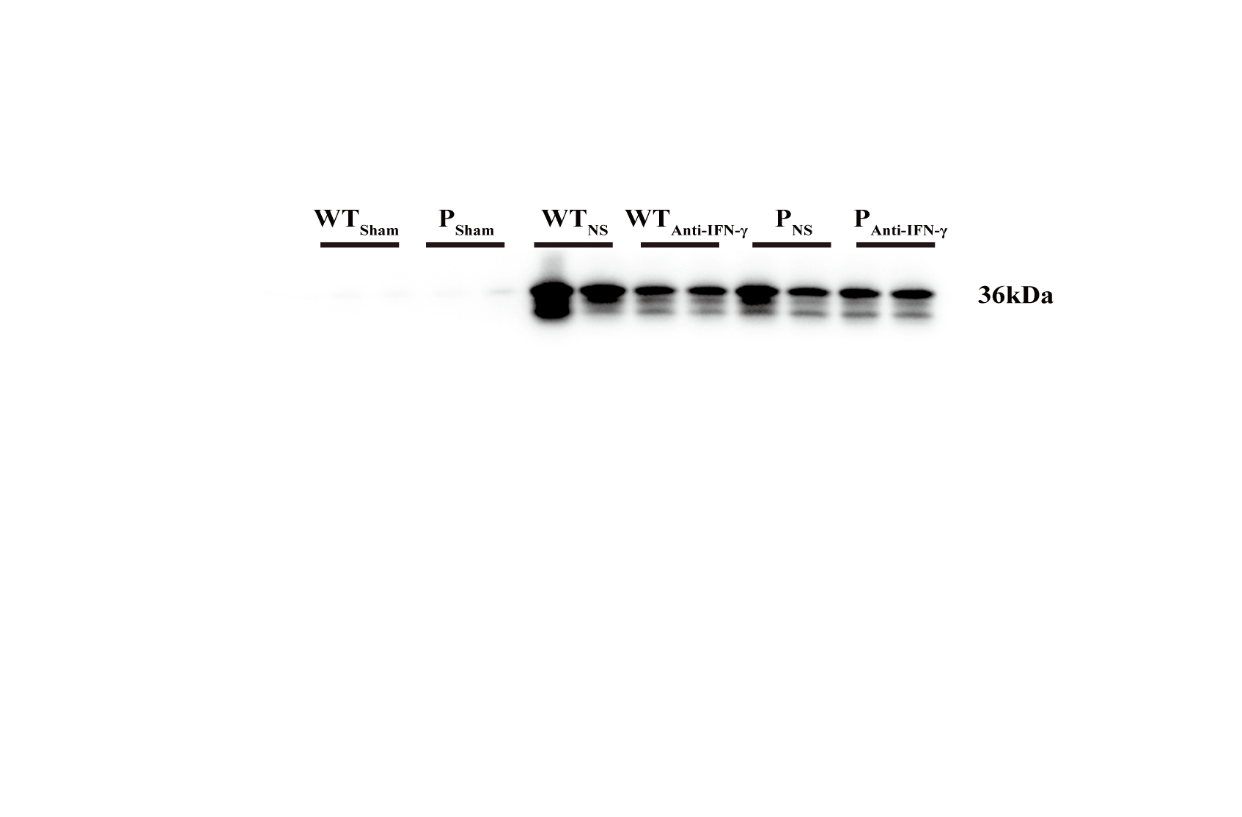

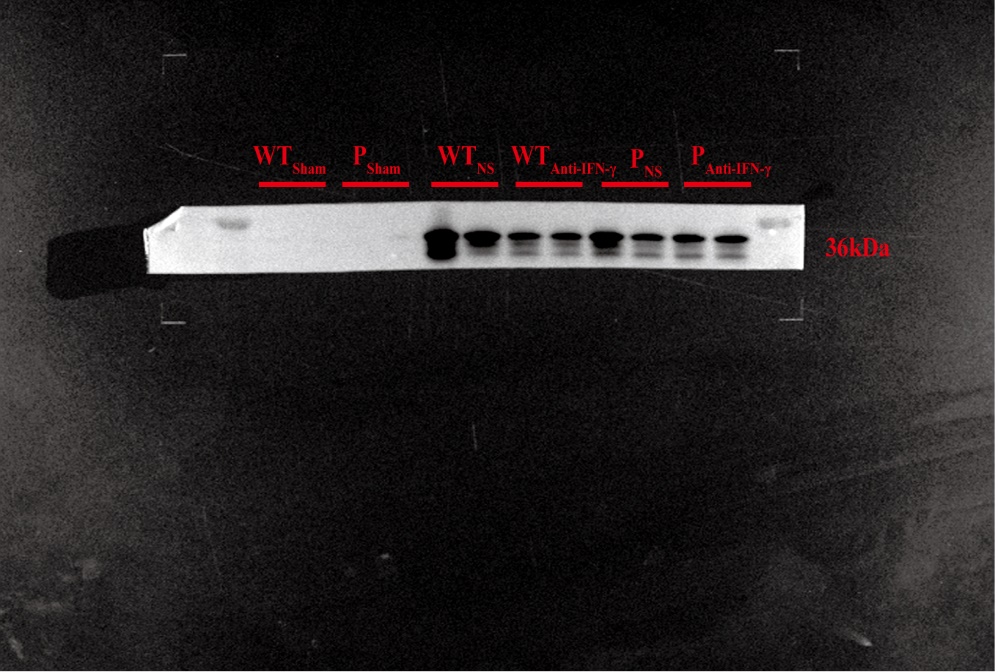


western blots of tubulin:

1.
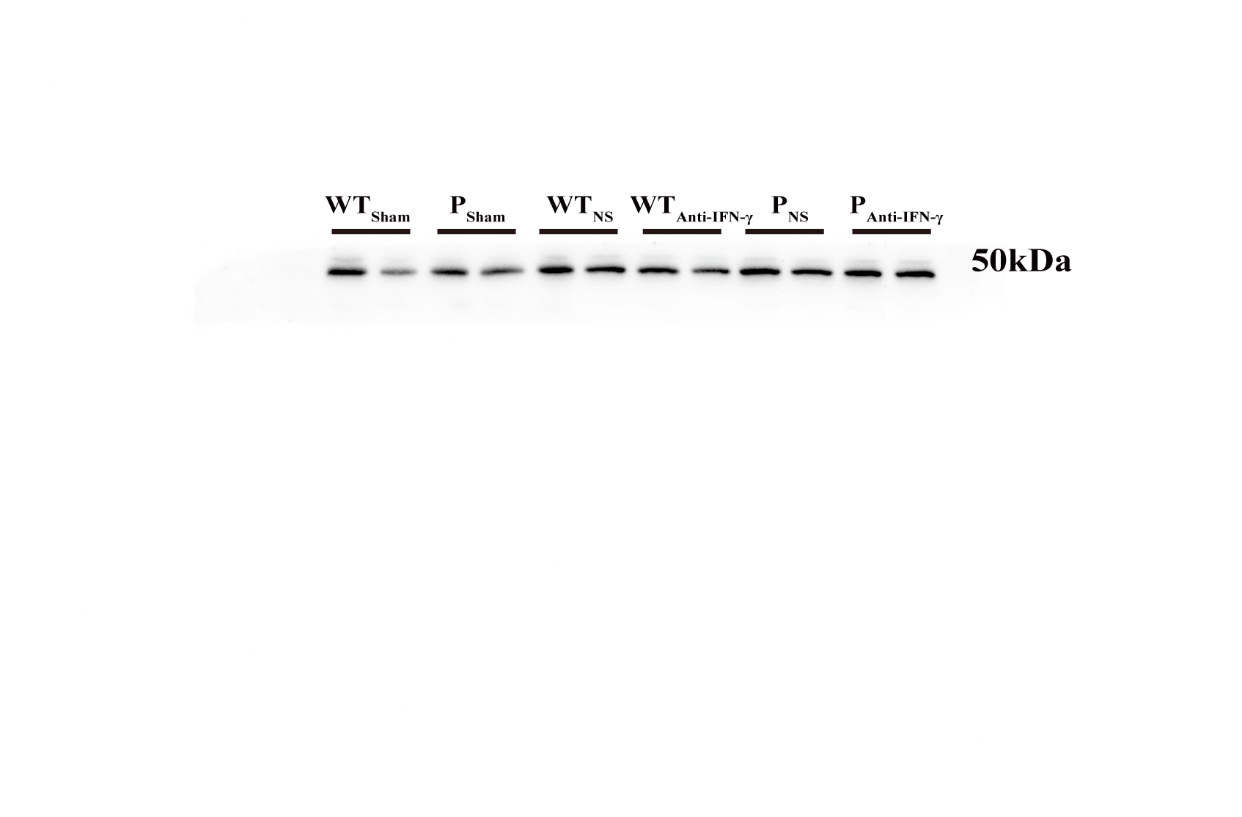

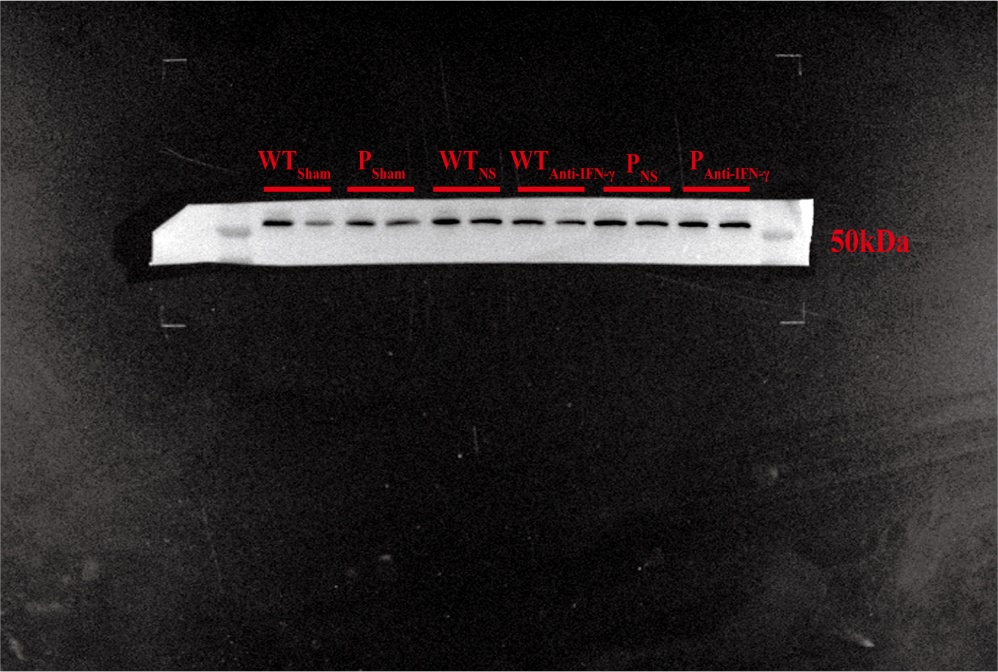


2.


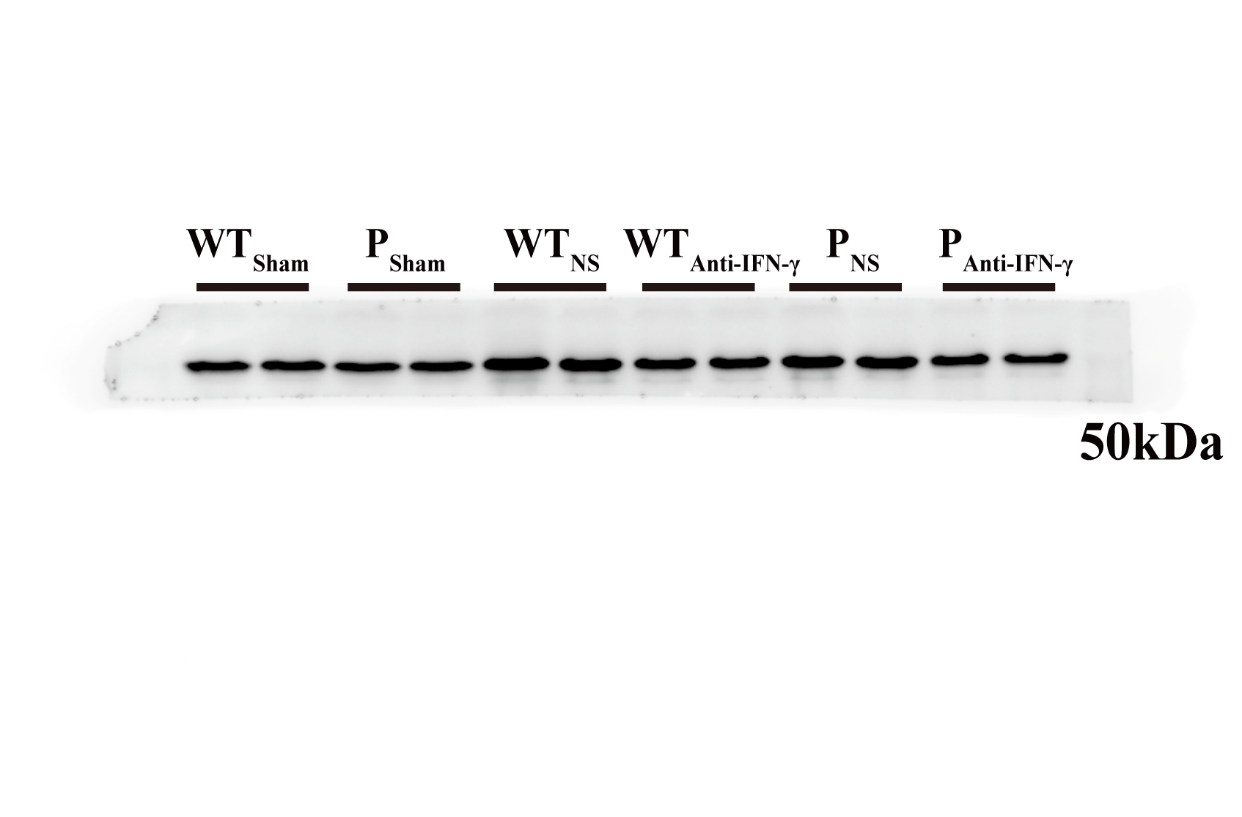

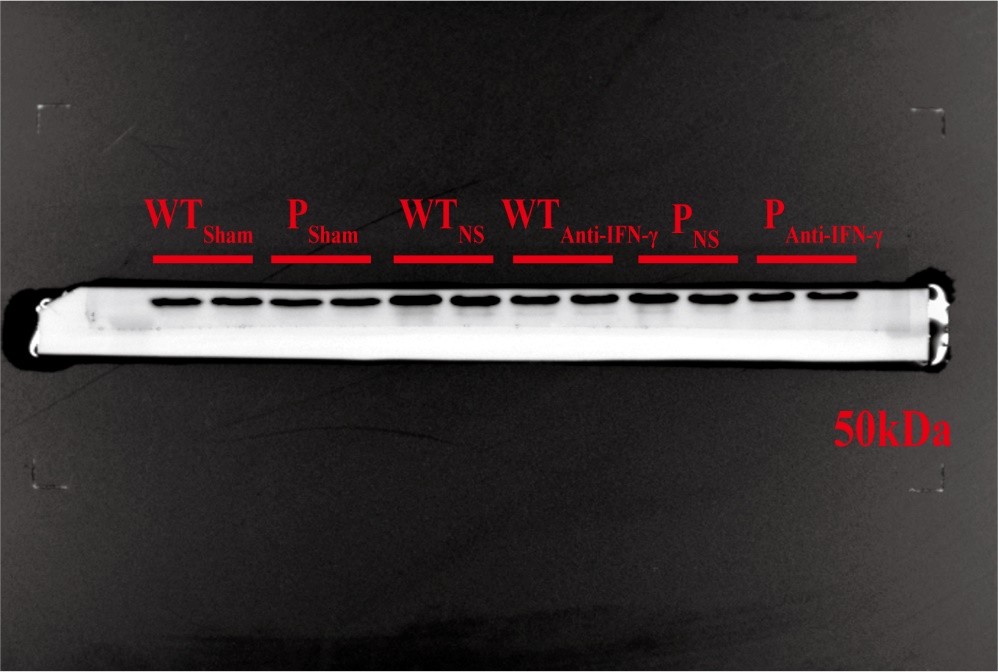

Supplement: Supplementary file 1 — Additional file 1. Original images of western blotting. [file 11658_2023_439_MOESM1_ESM.docx]
